# Neuropsychological function and past exposure to metallic mercury in female dental workers

**DOI:** 10.1111/j.1467-9450.2011.00929.x

**Published:** 2012-04

**Authors:** Helge Sletvold, Kristin Svendsen, Oddfrid Aas, Tore Syversen, Bjørn Hilt

**Affiliations:** 1 Private practiceTrondheim, Norway; 2 Department of Industrial Economics and Technology Management, Norwegian University of Science and TechnologyTrondheim, Norway; 3 Department of Occupational Medicine, St. Olavs University HospitalTrondheim, Norway; 4 Department of Neuroscience, Faculty of Medicine, Norwegian University of Science and TechnologyTrondheim, Norway; 5 Department of Public Health and General Practice, Faculty of Medicine, Norwegian University of Science and TechnologyTrondheim, Norway

**Keywords:** Visual memory, amalgam, urinary mercury, cognitive function

## Abstract

The aim of this study was to see if dental personnel with previous exposure to metallic mercury have later developed disturbances in cognitive function. Ninety-one female participants who had been selected from a previous health survey of dental personnel were investigated neuropsychologically within the following domains: motor function, short-term memory, working memory, executive function, mental flexibility, and visual and verbal long-term memory. The scores were mainly within normal ranges. Relationships between an exposure score, the duration of employment before 1990, and previously measured mercury in urine as independent variables and the neuropsychological findings as dependent variables, were analyzed by multiple linear regression controlling for age, general ability, length of education, alcohol consumption, and previous head injuries. The only relationship that was statistically significant in the hypothesized direction was between the previously measured urine mercury values and visual long-term memory, where the urine values explained 30% of the variability. As the study had a low statistical power and also some other methodological limitations, the results have to be interpreted with caution. Even so, we think it is right to conclude that neuropsychological findings indicative of subsequent cognitive injuries are difficult to find in groups of otherwise healthy dental personnel with previous occupational exposure to mercury.

## Introduction

The acute toxic effects of metallic mercury on CNS functions have been known for centuries. Symptoms of mercury poisoning comprise tremor, reduced psychomotor speed, paresthesia and a fairly distinct constellation of psychological symptoms, namely nervousness, shyness, blushing, irritability, and lability of mood. Mild forms of this syndrome are termed micromercurialism, whereas more grave conditions entailing psychotic symptoms are called erethism (Berlin, Zalups & Fowler, 2007; O’Carrol, Masterton, Dougall, Ebmeier & Goodwin, 1995). As a multitude of symptoms is observed, it is probable that the harmful effects of mercury involve various brain areas, such as cortical, subcortical and cerebellar.

Although the effects of acute mercury poisoning are well described and documented, the risk of long-term effects on the CNS from chronic low-level exposure is still debated. Previous studies of long-term effects from relatively low level exposures have been performed in different groups of mercury-exposed workers (Rohling & Demakis, 2006). In some Scandinavian countries, in particular in Norway and Denmark, the question of various late effects from mercury exposure in dental personnel has been a public issue during the last years.

Some studies of cognition and neuropsychological function in industrial workers exposed to mercury vapours have shown small, but statistically significant decreases in such domains as fine motor coordination, vigilance, psychomotor speed and visual memory (Ellingsen, Bast-Pettersen, Efskind & Thomassen, 2001; Mathiesen, Ellingsen & Kjuus, 1999), while other studies did not find any adverse effects on motor or cognitive tasks (Bast-Pettersen, Ellingsen, Efskind, Jordskogen & Thomassen, 2005; Zachi, Ventura, Faria & Taub, 2007). Two recent reviews with meta-analyses have concluded that long-term exposure to metallic mercury vapour with urine levels beyond 500 nmol/litre can lead to chronic effects on cognition, while there is more doubt as to whether lower exposures can lead to similar effects (Meyer-Baron, Schaeper, van Thriel & Seeber, 2004; Rohling & Demakis, 2006). From recent investigations it also seems evident that questions regarding individual susceptibilities play a significant role in the possible effects of metallic mercury exposure (Echeverria, Woods, Heyer *et al*., 2005).

In studies of the possible negative effects of mercury released from dental amalgam, no association between amalgam fillings and performance on tests of cognitive function was found in a homogenous group of elderly women (Saxe, Snowdon, Wekstein *et al*., 1995), and in two studies of patients with self-reported reactions to dental amalgam no difference was found between patients and a control group with regard to memory, attention and concentration (Dalen, Lygre, Kløve, Gjerdet & Askevold, 2003; Sundström, Bergdahl, Nyberg, Bergdahl & Nilsson, 2010).

Dental personnel have been exposed to metallic mercury when handling amalgam for teeth restoration. The degree of exposure has varied with the extent of the use, work performance and physical working conditions. In some countries, including Norway, a special kind of copper amalgam was used from the 1950s to the end of the 1980s for restoring deciduous teeth in particular. The procedure for preparing copper amalgam for use entailed the heating of solid tablets containing 70% metallic mercury for up to more than 200 °C. This could entail a substantial exposure to mercury vapor. Until the 1980s it was also customary for many of the assistants to handle amalgam in their bare hands in order to keep it soft while it was being used (Svendsen, Syversen, Melø & Hilt, 2010).

During the last decades there have been several studies that have shown various deleterious neuropsychological effects in current dental personnel in areas such as short-term memory (Uzzell & Oler, 1986), psychomotor speed, attention, cognitive flexibility and visual memory (Echeverria, Aposhian, Woods *et al*., 1998; Echeverria *et al*., 2005), visual scanning, verbal memory, visual memory and visuomotor coordination (Ngim, Foo, Boey & Jeyaratnam, 1992), vigilance (Ritchie, Gilmour, Macdonald *et al*., 2002), and verbal memory (Aydin, Karaoglanoglu, Yigit, Keles, Kirpinar & Seven, 2003). These studies have often been related to concurrent urine mercury levels. As far as we are aware, there has been only one previous study of dental personnel that has aimed at looking at possible relationships between past exposure to mercury and late cognitive function assessed by neuropsychological testing (Jones, Bunnell & Stillman, 2007). No difference was found between the exposed and control groups with regard to verbal memory and psychomotor speed and attention that were the only neuropsychological functions reported.

The aim of our study was to see if dental personnel with previous exposure to metallic mercury have later developed disturbances in such cognitive domains as psychomotor speed, attention, and verbal and visual long-term memory, which was hypothesized to be the case.

## Method

### Study participants

In a previous survey 856 female dental personnel under the age of 70 from central Norway had answered a standardized questionnaire called Euroquest that was developed to survey persons with neurotoxic exposures (Carter, Iregren, Söderman *et al*., 2002). For the present study we asked 120 of them living in the Trondheim area to participate in a neuropsychological examination. There were 26 who declined mainly due to subjective good health or because they considered their exposure as insignificant, and three who had to be excluded due to central nervous system illness (brain tumor or stroke), leaving the total number of participants in the present study as 91. To explore the representativeness of the 91 participants in relation to the group from which they were drawn, Table 1 shows age, sum of Euroquest symptom scores, a relative score of past cumulative exposure to mercury (Svendsen *et al*., 2010), as well as year of first employment and duration of employment adjusted for fraction of employment (Hilt, Svendsen, Syversen *et al*., 2009). Along with a somewhat higher age, those examined neurospsychologically also had an earlier start of employment and a higher relative exposure score, while the other features matched quite well between the groups.

**Table 1:** Distribution of some key features in 91 female dental personnel under the age of 70 examined neuropsychologically and in the group of 856 female dental personnel from which they were recruited

	Subsample examined neuropsychologically (*n *=* *91)				All women <70 years of age in original study (*n *=* *856)			
		
Feature	Mean	SD	Median	Range	Mean	SD	Median	Range
Age	57.0	6.4	58.0	33	50.5	10.8	52.0	48
Sum of Euroquest symptom scores^a^	11.9	3.2	11.2	14.2	11.9	3.2	11.2	14.2
Exposure score^a^	947	1,515	730	8,900	515	1084	168	11,245
Urine Hg value in nmol/l^b^	86.7	86.7	59.8	355	80.6	97.4	55.0	1068
Year of first employment^c^	1972	9.1	1971	42	1979	12.5	1977	51
Years of employment^c,d^	15.6	7.2	15.0	35.1	13.2	7.7	12.7	36.0

a Relative scores with no unit.

b 170 measurements available for all from previous survey, and 28 for those examined neuropsychologically in the current study.

c In dental health care.

d Before 1990 and adjusted for employment fraction.

### Mercury in urine

From 1955 up to 2000 there was a voluntary monitoring of mercury in urine for Norwegian dental personnel performed by the National Institute of Occupational Health with a total of 4,030 samples analysed for 2,028 persons (Lenvik, Woldbæk & Halgard, 2006). For 28 of the participants in the current study it was possible to gain access to their urine values which had been measured between 1962 and 1992 while they were still being exposed to metallic mercury during work in dental care. If a participant had more than one urine value available, the highest value was chosen. Data showing that the measured levels of mercury in urine matched quite well between the groups are also given in Table 1.

### Procedures

All examinations took place at the Department of Occupational Medicine, St. Olavs University Hospital in Trondheim, Norway. First, all participants were interviewed by one of three occupational physicians about their health conditions and their occupational history. In addition to information about the number of their own amalgam fillings and alcohol consumption from the questionnaire survey, the participants at the interview were asked about cerebral conditions, head injuries, cardiovascular diseases, hypothyreosis, epilepsy and diabetes mellitus. Blood pressure was measured and a venous blood sample was taken for the analysis of free thyroxin (FT4), thyreoidea stimulating hormone (TSH), alanine transaminase, cobolamin, folate, and serum creatinine. Each subject was tested by a clinical neurpsychologist (HS) on the same day as they were interviewed by one of the occupational physicians. When testing, the neuropsychologist did not know anything about the background data, health status or exposure status of the participants, and the same test procedure was followed for all. Depending on individual differences in working speed, the testing took from 1.5 to 2 hours, including a short break.

In order to be able to consider possible confounding from psychological complaints, the degree of subjective emotional symptoms was assessed by use of the SCL-90-R (Derogatis, 2009).

### Neuropsychological tests and cognitive function

In order to look at the general intellectual ability of the participants, we used the Similarities Test and the Picture Completion Test from the WAIS III (Wechsler, 2003) which measure verbal and non-verbal intelligence respectively.

The results from the particular neuropsychological tests used were combined into cognitive domains. Table 2 gives an overview of the domains and the different tests that were applied. Each test is described in further detail below.

**Table 2:** Neuropsychological domains and the tests used

Function	Tests
Motor function	Finger Tapping Test
Short-term memory	WAIS III, Digit Span Forwards
Working memory	WAIS III, Digit Span Backwards, PASAT 3 and 2
Executive function	Stroop and Two-choice Reaction Time With Inhibition (APT)
Mental flexibility	Trail Making B (TMT B) Minus A (TMT A)
Verbal long-term memory	California Verbal Learning Test II (CVLT-II)
Visual long-term memory	Doors Test part A and B

#### Motor function

The test used was the Finger Tapping Test from the Automated Psychological Test System APT) (Levander & Elithorn, 1987), and included tapping with the index fingers of both hands, alternation between the index and middle fingers of both hands, and alternation between right and left index fingers. The recorded score was the number of responses in a given time period for each condition for each hand, six scores in total.

#### Short-term memory

The Digit Span Forward from the WAIS III (Wechsler, 2003) was used, administered in the standardized way. The score used in this study was the maximum number of digits correctly repeated.

#### Working memory

Three tasks comprised this domain. In the Digit Span Backward (WAIS III), the subjects were asked to repeat the digits in the reverse order of presentation. Again, the score was the number of digits correctly repeated in reverse order.

In addition, two versions of the Paced Auditory Serial Addition Task (PASAT) were used (Gronwall & Wrightson, 1974). Taped series of 61 digits between 1 and 9 were presented to the subject. The task was to continuously add each digit to the digit preceding it. The responses were given verbally. In our version of the test, two rates of speed were used, namely 3 and 2 seconds, after an initial free response rate trial (Landrø, Celius & Sletvold, 2004). The scores were the number of correct responses for each presentation rate.

#### Executive function

Two tasks were included, one subtest from the Stroop test (Lund-Johansen, Hugdahl & Wester, 1996) and the Two-choice Reaction Time with Inhibition (APT) (Levander & Elithorn, 1987). In the Stroop subtest (Stroop word/colour), the task was to inhibit the overlearned tendency to read. Two scores were reported: the time used and the number of incorrect responses.

In the APT test, the subject was required to inhibit her response to a visual stimulus when presented together with a sound, but not otherwise (Go-NoGo). Two scores were calculated. One was the mean reaction time in milliseconds for each hand respectively, and the other was the number of failed inhibitions.

#### Simple and choice reaction time

The tests included were the simple auditory, simple visual and two-choice reaction time tests, all from the APT. All measures were given in milliseconds.

#### Mental flexibility

This score was computed using the difference between the Trail Making Test B (TMT B) and Trail Making Test A (TMT A). This was done to correct for baseline motor performance (Landrø, Stiles & Sletvold, 2001). Standard administration procedures were followed. The score was the time difference in seconds between the TMT B and TMT A for each subject.

#### Verbal long-term memory

The California Verbal Learning Test II (CVLT-II) was used (Delis, Kramer, Kaplan & Ober, 2004). The test was administered in a standardized way. The following scores were reported: free recall after each presentation (CVLT A1-5), sum of recalled words across the five presentations (sum CVLT A1-5), free recall after distraction (CVLT IR), immediate cued recall (CVLT ICR), delayed recall (CVLT DR), recognition (CVLT recognition), false positive responses (CVLT FP), and intrusions (CVLT intrusion).

#### Visual long-term memory

The Doors Test (Baddeley, Emslie & Nimmo-Smith, 1994) is a task of visual recognition in two versions, one easy (Doors A) and one difficult (Doors B). In each version, the subject was presented with 12 target doors consecutively, each target door being exposed for about 3 seconds. Thereafter, each of the 12 target doors was presented together with three doors not previously seen, the task being to identify the correct (target) door. Parts A and B were both scored separately and as a sum score of A+B.

### Other measures

#### Hand tremor

Tremor was recorded using the Tremor Pen (Catsys, 2000). Tremor was registered for 8 seconds for each hand respectively. There are four different tremor measures: Tremor Intensity, defined as the root mean square of acceleration, measured in m/s^2^. Center Frequency, measured in Hz, is the mean of the accelerations in the frequency area 0.9 to 15.0 Hz during the registration period. The standard deviation of the Center Frequency concerns the degree of coordination of the tremor. Lastly, the Harmonic Index refers to a comparison between the frequency pattern of the tremor and the pattern of one single oscillation.

### Statistical methods

Data were registered and analyzed with the data program Statistical Package for Social Science version 17.0 (SPSS) (SPSS Inc., Chicago, IL, USA). Because the scores for each particular neuropsychological test could be on different scales and could have both positive and negative values, the test scores were transformed into *z*-scores based on means and standard deviations from the current sample before they were added together in the seven different cognitive domains. Possible relationships between the three chosen independent exposure variables (the total exposure scores, the number of adjusted years of employment in dental care before 1990, and the previously measured urine levels) and the dependent variables (the cognitive domains) were analyzed by multiple linear regression. For exploration of possible confounding factors, and to build models for the final analyses, we first used a model with age, general ability, length of education, number of own amalgam fillings, alcohol consumption, systolic blood pressure, the occurrence of cardiovascular disease, epilepsy, hypothyreosis, previous head injuries, and the SCL-90-R depression score (Tables 1 and 2) as independent variables in a stepwise multiple regression with the different cognitive functional domains as dependent variables. Those independent variables that had a statistically significant effect on at least one of the dependent variables were included in three final models with forced entry of the three chosen independent exposure variables one at a time, along with age, general ability, length of education, alcohol consumption, previous head injuries, and SCL-90-R as possible confounders. A 5% level was chosen for statistical significance, and all *p*-values were calculated as two-sided.

Mean values of the neuropsychological tests were compared either to existing norms or relevant normal controls from other studies.

### Ethical considerations

The study was approved by the ethical committee for medical research in Central Norway, and we had a license for personal registrations from the Norwegian Social Science Data Services. The conduct of the study was deemed to be in accord with the Helsinki declaration on medical research ethics.

## Results

Among the 91 participants there were 16 dentists, 69 dental assistants, and 6 who belonged to other occupational groups within dental health care. Table 3 shows the distribution of some continuous background variables, general intellectual ability, and the SCL-90-R depression and global symptom index scores. Compared to Norwegian norms for females, the scores are well within normal ranges (Vassend, Lian & Andersen, 1992). Test scores for general intellectual ability were also within normal ranges. Table 4 shows the occurrence of some lifestyle factors, current diseases, and previous head injuries in the 91 participants. Hypertension was defined as systolic blood pressure over 140 or diastolic over 90 respectively. The reported head injuries were mostly moderate cases of concussion with only little or no unconsciousness. None of the participants reported suffering from diabetes mellitus.

**Table 3:** Background variables for 91 dental assistants with previous exposure to metallic mercury in dental health care

Variable	Min	Max	Mean	SD
Age	34	67	57	6.4
Years of education	8	21	13.1	3.2
Number of own amalgam fillings	1	28	9.6	5.1
WAIS III Similarities	4	17	9.7	2.8
WAIS III Picture Completion	6	16	11.1	2.2
SCL-90-R Depression score	0	1.8	0.6	0.4
SCL-90-R GSI	0	1.7	0.4	0.3

**Table 4:** Lifestyle factors, current diseases, and head injuries in 91 dental assistants with previous exposure to metallic mercury in dental health care

Life style factors and diseases / injuries	Prevalence in %
Smoking	
Never	45.1
Previous smoker	38.5
Occasionally	5.5
Daily smoker	11.0
Alcohol consumption during the last year	
No	3.3
Yes, but more seldom than every week	53.8
Yes, every week	42.9
Diseases / injuries	
Cardiovascular disease	19.8
Hypertension	31.9
Epilepsy	1.1
Hypothyreosis	12.1
Previous head injury	13.2

Blood samples, which were analyzed for free thyroxin (FT4), thyreoidea stimulating hormone (TSH), alanine transaminase, cobolamin, folate, and serum creatinine, revealed values within normal ranges for all tests in all individuals. This gave an indication that the participants did not have other diseases that could impair their cognitive function, and that the eleven subjects who suffered from hypothyroidism seemed well regulated.

Table 5 shows the raw scores from each of the neuropsychological tests performed on the participants, as well as raw scores yielding average standardized scores and comparable reference values from healthy control groups in other studies (Baddeley *et al*., 1994; Bast-Pettersen, 2006; Delis *et al*., 2004; Egeland, Sundet, Landrø*et al*., 2005; Landrø, Stiles & Sletvold, 1997; Rasmussen, Levander & Sletvold, 1995; Sletvold, Stiles & Landrø, 1995; Spreen & Strauss, 1991). When we compared the mean values with existing norms for the corresponding age groups and normal control group data from other studies, we found that 29 out of 41 results were within one standard deviation from the normal mean, while seven scores, all from motor and reaction time tests, were below the normal range. Five other tasks, tagged with asterisks in the table, lacked norms or relevant control group data.

**Table 5:** Raw scores for particular neuropsychological tests and reference values in female dental workers with occupational exposures to metallic mercury

	Raw scores for participants				Reference values		
			
Test	Min	Max	Mean	SD	Mean	SD	Score >1 SD below reference mean
Wais III digit span forwards	4	10	5.9	1.1	6.4^a^	0.7	
Wais III digit span backwards	3	8	4.6	1.2	5.1^a^	0.8	
Trail making test A	18.0	83.8	36.4	12.5	32.2^b^	8.1	
Trail making test B	42.2	172.7	82.2	25.3	63.9^b^	15.4	
Trail making test B incorrect	0	6	0.4	0.9	*		
Trail making test B-A	−5.7	121.8	45.8	21.8	*		
PASAT3 correct	19	60	42.7	11.0	52.0^c^	8.7	
PASAT3 incorrect	0	18	5.5	3.8	*		
PASAT2 correct	14	51	30.5	8.8	35.6^d^	14.6	
PASAT2 incorrect	0	15	4.7	2.8	*		
Finger tapping right	3.95	8.14	5.8	0.9	6.6^e^	0.8	#
Finger tapping left	3.49	8.02	5.5	0.9	6.3^e^	0.8	#
Finger tapping alt.right	1.06	4.58	2.7	1.1	4.2^e^	1.1	#
Finger tapping alt.left	1.01	4.48	2.6	0.9	4.0^e^	0.9	#
Finger tapping alt.right/left	1.30	4.59	3.1	0.8	4.6^e^	0.9	#
Reaction time audi	161.1	462.9	230.3	44.8	213^e^	71	
Reaction time visu	170.9	441.0	242.0	39.0	211^e^	42	
Reaction time choice right	210.6	400.5	292.0	44.5	263.7^b^	36.0	
Reaction time choice left	205.2	428.2	295.1	40.1	254.3^b^	37.2	#
Reaction time inhib.right	293.8	817.5	515.5	106.3	487.5^b^	118.8	
Reaction time inhib.left	293.8	971.8	624.6	123.2	475.8^b^	100	#
Reaction time failed inhib	0	4.8	1.1	1.1	1.5^e^	2.9	
Stroop color	16.10	42.40	26.8	4.8	33.5^f^	8.6	
Stroop words	10.40	24.30	17.3	2.6	20^f^	3.9	
Stroop word/color	33.6	112.3	53.5	14.1	74.2^f^	22.5	
Stroop word/col incorr	0	13	1.8	2.6	*		
CVLT A1	3	15	6.5	2.1	7^g^		
CVLT A2	6	16	10.2	2.5	9^g^		
CVLT A3	6	16	11.6	2.5	11^g^		
CVLT A4	9	16	12.8	2.0	12^g^		
CVLT A5	9	16	13.5	1.9	13^g^		
Sum CVLT A1-5	34	79	54.5	9.4	49–50^g^		
CVLT IR	5	16	11.7	2.7	11^g^		
CVLT ICR	7	16	12.4	2.4	12^g^		
CVLT DR	8	16	12.7	2.2	11–12^g^		
CVLT recognition	11	16	15.2	1.1	15^g^		
CVLT FP	0	11	1.1	1.9	2^g^		
CVLT intrusions	0	10	2.9	2.2	3–4^g^		
DoorsA raw score	6	12	10.9	1.4	11^h^		
DoorsB raw score	2	12	7.3	2.0	7–8^h^		
DoorsAB raw score	11	24	18.2	2.8	18–19^h^		

* No available norms.

a Landrø*et al*., 1997, healthy controls with mean age 40.1 (9.6)

b Sletvold *et al.*, 1995, healthy controls with mean age 40.1 (9.6)

c Egeland *et al.*, 2005, Norwegian norms for ages 35–44.

d Spreen & Strauss, 1991, Norms for ages 50–69.

e Rasmussen *et al*., 1995, Controls for ages 20–45.

f Bast Pettersen, 2006, Norms for ages 55–61.9.

g Delis *et al.*, 2004, Raw scores for the corresponding age group yielding a *z*-score of 0.0, except for CVLT 1-5 which gives the raw scores corresponding to a *T*-score of 50.

h Baddeley *et al.*, 1994, Raw scores for the corresponding age group yielding a scaled score of 10.

**Fig. 1:**
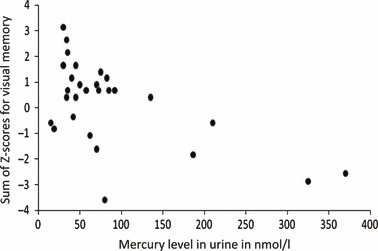
Maximum measured mercury urine values in relation to visual memory.

Table 6 shows the results of the forced entry linear regression analysis with the total exposure score, years of employment in dental care prior to 1990, and the maximum previously measured urine mercury value as the determinants with adjustments for age, general ability, length of education, alcohol consumption, previous head injuries, and SCL-90-R depression score in the model applied. For all domains, apart from executive function and mental flexibility, a higher value was indicative of a better function. That is, that a negative beta (β) was indicative of a result in the hypothesized direction. There was a statistically significant inverse relationship between the total exposure score and motor function and between the number of years worked in dental health care before 1990 and long-term visual memory. The only relationship that was statistically significant in the hypothesized direction was between the previously measured urine mercury values and visual long-term memory, where the urine values explained 30% of the variability. Figure 1 presents a scatter-plot of this relationship. It is shown that there are mainly four or five results that make out the difference.

**Table 6:** Forced entry linear regression analysis of relationships between the total exposure score, the number of years of employment before 1990, and previously measured mercury in urine and the cognitive functional domains adjusted for age, general ability, length of education, alcohol consumption, the SCL-90-R depression score, and previous head injuries

	Motor function			Short-term memory			Working memory			Executive function			Mental flexibility			Verbal long-term memory			Visual long-term memory		
							
Determinants	β	p	R^2*^	β	p	R^2^	β	p	R^2^	β	p	R^2^	β	p	R^2^	β	p	R^2^	β	p	R^2^
Exposure score	0.21	0.05	0.11	−0.04	0.73	0.06	0.01	0.95	0.11	−0.07	0.48	0.21	−0.05	0.65	0.23	0.67	0.51	0.15	−0.13	0.23	0.12
Years work before 1990	0.19	0.15	0.09	−0.07	0.62	0.06	0.05	0.70	0.11	−0.13	0.27	0.21	−0.10	0.39	0.23	0.13	0.29	0.16	0.34	0.01	0.18
Max urine Hg value	−0.01	0.97	0.07	0.07	0.74	0.06	−0.28	0.21	0.35	0.12	0.54	0.37	0.14	0.54	0.09	−0.07	0.77	0.03	−0.58	0.01	0.30

* Amount of explained variation by the model.

## Discussion

The only hypothesized relationship between the indicators of mercury exposure and cognitive function that was found was in the regression analysis of a relationship between previously measured urinary mercury and long-term visual memory. Other exposure indicators, such as our calculated total exposure score and the length of employment in dental care prior to 1990, did not show any statistically significant relationship apart from a few that went in the inverse direction.

As the participants were examined without knowledge about their health status or previous exposure situation, and relevant potential confounding factors like age, length of education, alcohol consumption, and previous head injuries were controlled for in the analyses, bias probably does not provide an explanation for the mainly negative results. In that regard it is somewhat remarkable that 12.1% of the participants reported having hypothyreosis. There can be different reasons for that, but one hypothesis that should be looked into is that the occurrence of hypothyreosis is increased in females who have been exposed to mercury. As the distribution of Euroquest scores was similar, we also believe that the subgroup was a representative selection from our initial study group. The distribution of the exposure scores in the initial study and this study also indicates that there was no serious selection bias in the neuropsychological study. One small bias might, however, have been introduced as the 26 who declined participation mainly did so because they were without any complaints and/or because they assessed their own exposure as insignificant. In regard to the neuropsychological methods that were applied, we are confident that they were relevant and sufficiently sensitive as such.

One problem with the present study is, however, its low statistical power. In the primary questionnaire survey on which the present study is based, we revealed prevalences in female dental assistants of between 0.4% and 2.8% of assumed cognitive malfunction related to occupational exposure to metallic mercury (Hilt *et al*., 2009). Even though self-reported data cannot be compared to more objective test data, it would have been useful if these findings had been available at the time when the present study was designed and performed. With the low prevalences of cognitive symptoms in the study base, and an assumed low to moderate exposures to metallic mercury in dental personnel (Lenvik *et al*., 2006; Svendsen *et al*., 2010), it can hardly be expected to find any differences in neuropsychological performance by available methods on a group basis unless the number of subjects is considerably extended. In that light, the observed relationship between historical urine mercury levels and impairments in visual memory in a small subsample of our participants is somewhat remarkable. Positive relationships between current low level urinary mercury and measures of cognitive function have previously been shown in some investigations (Echeverria, Heyer, Martin, Naleway, Woods & Bittner, 1995; Ritchie, Macdonald, Hammersley *et al*., 1995) but, as far as we are aware, not for historical urine values. However, the mercury in urine measurements that had been taken from 28 of the participants had not been taken systematically and did not necessarily reflect their true cumulative exposure to metallic mercury. And, as there are individual differences in both mercury toxicokinetics and susceptibility, there does not need to be any strong correlation between a single urine mercury level and a disease outcome. The observed apparent relationship may of course also be a statistical coincidence due to multiple testing, but as all the investigated domains apart from short-term memory, went in the hypothesized direction, we do not consider that to be very likely. A Bonferroni adjustment could have been appropriate, but as the outcomes were related, and the results were marginal, this was abandoned.

Another feature that may have influenced our results is that individual susceptibilities may be equally important determinants for chronic cognitive effects as the level of exposure as such (Echeverria *et al.*, 2005; Echeverria, Woods, Heyer, Rohlmann, Farin & Garabedian, 2006; Heyer, Echeverria, Bittner, Farin, Garabedian & Woods, 2004). At the time when the study was planned, we had no means of measuring differences in individual susceptibility.

Previous studies of adverse effects of metallic mercury in currently exposed dental personnel have shown moderate effects on cognitive function (Aydin *et al*., 2003; Echeverria *et al*., 1995; Ngim *et al*., 1992; Ritchie *et al*., 1995, 2002; Uzzell & Oler, 1986.) However, the clinical significance of the findings can be questioned. Our observation of no relationships based on an assessment of cumulative previous exposure is therefore not contradictory to these findings. As we also question the clinical significance of the observed relationship between urinary mercury levels and cognitive function, our results do not reject previous conclusions that state that there is little evidence for long-term cognitive dysfunction caused by exposures with urine mercury levels under 50 μg/l (about 500 nmol/l) (Rohling & Demakis, 2006). It can also be argued that our study should have included an entirely unexposed control group. Comparing our results with available norms and control group data, we found that 71% of the results were within one standard deviation from the mean reference values. Taking into consideration that motor and reaction time tasks are sensitive to aging (Lezak, Howiesen & Loring, 2004), and our study group is older than the groups from which the reference values have been derived, it is probable that this share would be even higher. We also claim that our study group had a fair and representative distribution on the determinant scale, including subjects with relatively low exposures.

Another limitation in regard to the generalizability of the present study is that men were not included. There were only 227 men under the age of 70 who had participated in the initial survey. The low number would have made a stratified analysis impossible. Moreover, most of the participating men were dentists with higher education and with relatively low exposures, which could also have led to confounding as a result of different educational levels.

Our finding of a relationship between previously measured urinary mercury values and a reduction in visual long-term memory is of particular interest from a neuropsychological point of view. In our study, visual memory was investigated using a recognition task with two levels of difficulty. The reduced performance was evident on both the easy and difficult parts of the Doors test (Table 5), indicating a rather stable effect. A well-functioning visual memory relies on intact sensory, perceptual, storing and retrieval capacities, involving pathways to areas in the occipital, parietal and temporal cortices, as well as the hippocampus.

Some studies have shown impairments in color discrimination and contrast sensitivity in subjects exposed to metallic mercury (Canto-Pereira, Lago, Costa *et al*., 2005; Feitosa-Santana, Costa, Lago & Ventura, 2007). It has been hypothesized that the entire visual system may be compromised in a diffuse way by the toxic effects of mercury (Goldman-Rakic & Leung, 2002). Previous neuropsychological studies have shown reduced visual memory and visuospatial function in dental personnel and mercury-exposed industrial workers (Echeverria *et al*., 1995; Feitosa-Santana *et al*., 2007), while other studies have found no impairment in these cognitive domains (Bast-Pettersen *et al*., 2005; Zachi *et al*., 2007). In general, visuospatial processing and memory are two of the domains where one of the previous meta-analyses has concluded with small, but significant effects (Rohling & Demakis, 2006). Our results should be treated with caution, however, as the relationship between urine mercury levels and visual memory is based on only a few cases with relatively high exposures (Fig. 1). When exploring the data, three out of five had more than one measurement with urinary mercury beyond 100 nmol/l making it less probable that the higher values represent outliers. Among the 2,028 dental personnel who had previous urine monitoring performed by the National Institute of Occupational Health in Norway, 2.5% had mercury values beyond 300 nmol/l (Lenvik *et al.*, 2006).

Because of the restrictions and limitations mentioned, any interpretation of the results has to be made with caution. Even so, we find that our investigation has been carefully performed with little selection bias and a reasonable control of possible confounding factors.

## Conclusion

Based on our study and available knowledge, it seems reasonable to conclude that neuropsychological findings indicative of subsequent cognitive injuries can be difficult to find in groups of otherwise healthy dental personnel with previous exposure to metallic mercury. For further studies, we think that these have to be undertaken on subjects with both relevant cognitive complaints and sufficient exposures, and preferably, where features of individual susceptibility are also taken into account.
